# Use of Remote Camera Traps to Evaluate Animal-Based Welfare Indicators in Individual Free-Roaming Wild Horses

**DOI:** 10.3390/ani11072101

**Published:** 2021-07-15

**Authors:** Andrea M. Harvey, John M. Morton, David J. Mellor, Vibeke Russell, Rosalie S. Chapple, Daniel Ramp

**Affiliations:** 1 Centre for Compassionate Conservation, School of Life Sciences, University of Technology Sydney, Ultimo, NSW 2007, Australia; Daniel.Ramp@uts.edu.au; 2 Jemora Pty Ltd., P.O. Box 2277, Geelong, VIC 3220, Australia; johnmorton.jemora@gmail.com; 3 Animal Welfare Science and Bioethics Centre, School of Veterinary Science, Massey University, Palmerston North 4442, New Zealand; D.J.Mellor@massey.ac.nz; 4 Veterinary Contractor, c/o Animal Emergency Australia, P.O. Box 1854, Springwood, QLD 4217, Australia; vibekeanna@yahoo.com.au; 5 Blue Mountains World Heritage Institute, 16 Dunmore Lane, Katoomba, NSW 2780, Australia; r.chapple@bmwhi.org.au

**Keywords:** welfare assessment, animal-based welfare indicators, camera traps, wild horses

## Abstract

**Simple Summary:**

Knowledge of the welfare status of wild animals is critical for informing debates about how we interact with them. However, methodology to enable assessment of the welfare of free-roaming wild animals has been lacking. In this study, we assessed the use of remote camera traps to non-invasively identify individual free-roaming wild horses and evaluate an extensive range of welfare indicators. Camera trapping was successful in detecting and identifying horses across a range of habitats including woodlands where horses could not be directly observed. Twelve indicators of welfare were assessed with equal frequency using both still images and video, with an additional five indicators assessed on video. This is the first time such a methodology has been described for assessing a range of welfare indicators in free-roaming wild animals. The methodology described can also be adjusted and applied to other species, enabling significant advances to be made in the field of wild animal welfare.

**Abstract:**

We previously developed a Ten-Stage Protocol for scientifically assessing the welfare of individual free-roaming wild animals using the Five Domains Model. The protocol includes developing methods for measuring or observing welfare indices. In this study, we assessed the use of remote camera traps to evaluate an extensive range of welfare indicators in individual free-roaming wild horses. Still images and videos were collected and analysed to assess whether horses could be detected and identified individually, which welfare indicators could be reliably evaluated, and whether behaviour could be quantitatively assessed. Remote camera trapping was successful in detecting and identifying horses (75% on still images and 72% on video observation events), across a range of habitats including woodlands where horses could not be directly observed. Twelve indicators of welfare across the Five Domains were assessed with equal frequency on both still images and video, with those most frequently assessable being body condition score (73% and 79% of observation events, respectively), body posture (76% for both), coat condition (42% and 52%, respectively), and whether or not the horse was sweating excessively (42% and 45%, respectively). An additional five indicators could only be assessed on video; those most frequently observable being presence or absence of weakness (66%), qualitative behavioural assessment (60%), presence or absence of shivering (51%), and gait at walk (50%). Specific behaviours were identified in 93% of still images and 84% of video events, and proportions of time different behaviours were captured could be calculated. Most social behaviours were rarely observed, but close spatial proximity to other horses, as an indicator of social bonds, was recorded in 36% of still images, and 29% of video observation events. This is the first study that describes detailed methodology for these purposes. The results of this study can also form the basis of application to other species, which could contribute significantly to advancing the field of wild animal welfare.

## 1. Introduction

Knowledge of the welfare status of wild animals is critical for informing ethical, legal, and political debates about the ways we interact with them. The importance of developing scientific methods for capturing data to enable the assessment of the welfare of free-roaming wild animals has recently been highlighted [1]. Many studies have evaluated wild horse behaviours, time budgets, home ranges, body condition scores and social organisation, e.g., [2,3,4,5,6,7,8,9,10,11,12,13,14,15,16,17,18,19], but to date, an extensive range of welfare indicators has apparently not been assessed.

We recently published a Ten-Stage Protocol for scientifically assessing the welfare of individual non-captive wild animals, using free-roaming horses as an example [1]. Table 1 lists these ten stages.

The protocol uses the Five Domains Model for assessing animal welfare [20,21,22,23,24]. The Five Domains Model comprises four interacting physical/functional domains of welfare—‘Nutrition’, ‘Physical Environment’, ‘Health’ and ‘Behavioural Interactions’—and a fifth domain of ‘Mental State’. Following measurement of animal-based indices within each physical/functional domain, the anticipated negative or positive affective consequences are cautiously assigned to Domain 5. Three of the ten stages of the Ten-Stage Protocol relate specifically to the Model: Stage 4, develop a comprehensive list of potential measurable or observable indicators of welfare in each physical/functional domain; Stage 5, select methods to reliably identify individual animals; and Stage 6, select methods for measuring or observing potential welfare indices, then evaluate which indices can be practically measured or observed in the specific context of the study. This last stage requires further investigation to identify which indices can be measured or observed using the selected methods, before detailed studies to assess welfare can be conducted. That is the primary focus of this paper.

Current knowledge of wild horse ecology and behaviour has historically relied on direct observations of horses, e.g., [2,3,4,5,6,7,8,9,10,11,12,13,14,15,16,17,18,19]. The disadvantages of direct observations for assessing indicators of welfare include practical limitations on the number of observations of individual animals that can be made, and usually the need to observe them from long distances. This may make evaluation of some welfare indicators challenging, and it restricts observations to only those animals that can be seen directly, primarily in open areas. Horses residing in woodland habitats and/or challenging terrain, or those that become separated from their band, for example due to debilitation or injury, are less likely to be observed directly and hence may be underrepresented in resulting datasets.

Camera traps have been widely used to study wildlife in a variety of habitats, e.g., [25,26,27,28], focusing on a range of variables such as abundance and density, e.g., [29], and demographic measures, e.g., [30,31]. More recently, they have been used to collect behavioural data from a range of captive and wild animals, e.g., [32,33,34,35] and to evaluate leprosy-like skin lesions in wild chimpanzees [36]. They have also been used to collect data on some aspects of behaviour of wild Przewalski horses [37,38]. However, to date, there are apparently no published reports of camera trap use directed specifically at evaluating a comprehensive range of animal-based welfare indicators in any mammalian species, whether captive or free-roaming.

The aims of this study were to evaluate the use of camera traps, both still images and video, across a range of habitats, to assess (a) whether free-roaming wild horses could be detected and individually identified, (b) which animal-based welfare indicators could be practically and reliably evaluated, (c) the reasons for not being able to identify horses or assess welfare indicators, and (d) whether behaviour could be quantitatively assessed. We specifically describe our methodological information in detail in order to enable other researchers to replicate these methods. Furthermore, we sought to identify advantages and limitations of the use of camera traps for these purposes.

## 2. Materials and Methods

### 2.1. Study Overview

We made camera trap and direct observations of a small and geographically constrained population of free-roaming wild horses in Australia over a 15 month period.

#### 2.1.1. Selection of Welfare Indicators

In line with Stage 4 of the published Ten-Stage Protocol (Table 1) for assessing welfare [1], we developed an extensive list of potential measurable/observable indicators in each physical/functional domain, based on a literature search (using the terms ‘horse welfare’, ‘equine welfare’, ‘welfare indicators’) for previously reported animal-based welfare indicators in domestic horses [13,39,40,41,42,43,44,45,46,47,48,49,50,51,52,53,54,55,56,57,58,59]. As already noted, the physical/functional domains of the model are: 1. Nutrition; 2. Physical Environment; 3. Health; and 4. Behavioural Interactions [24]. From this list, indicators were divided into specific measures of current welfare status, and alerting measures that draw attention to potential welfare risks [1]. We then selected measurable or observable welfare indicators that may be able to be captured in free-roaming horses using camera trap still images and/or videos or by direct observation (Table 2).

#### 2.1.2. The Study Area and Horse Population

**Figure 1:**
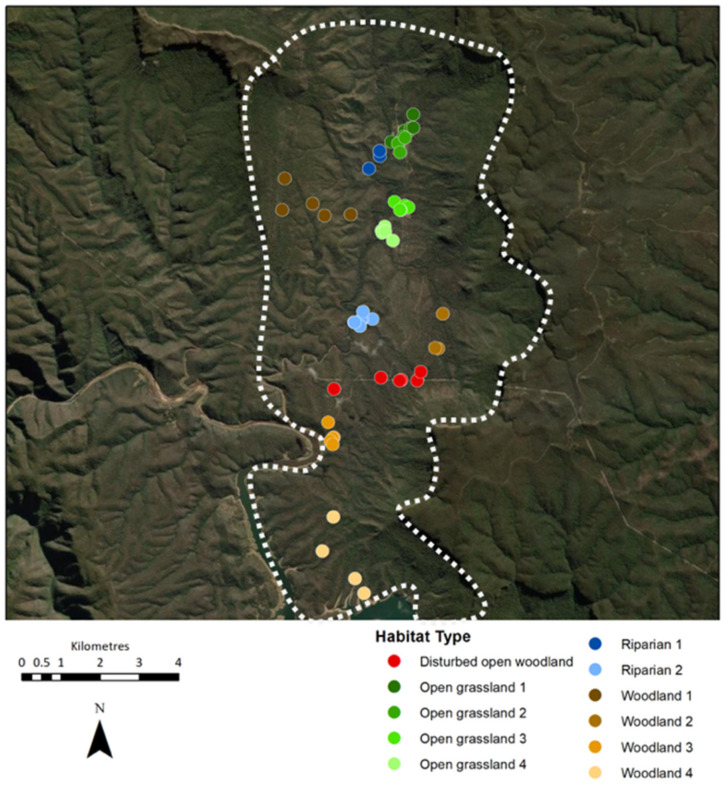
Topographical map of the study area illustrating camera locations and habitat types in the 11 areas. The white dashed line represents the geographic boundaries that inhibit immigration and emigration of this horse population.

**Figure 2:**
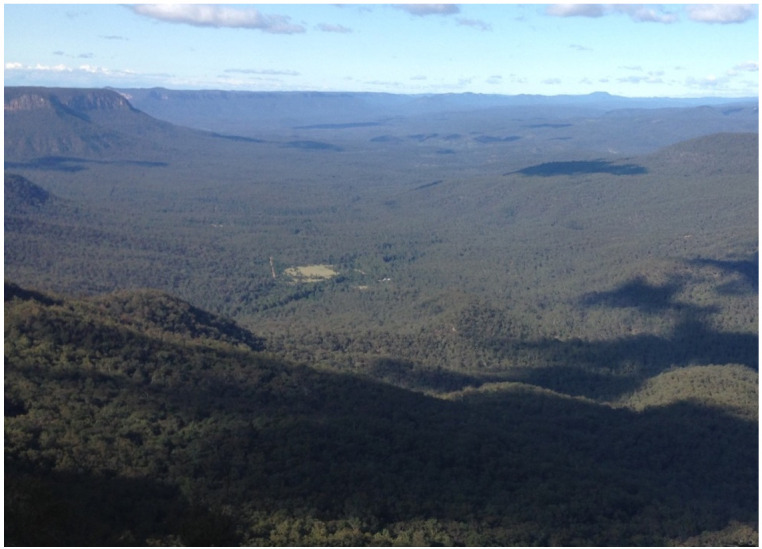
View of Kedumba valley facing south. The grassland areas visible near the middle of the picture are the regions named Open grassland 1. and 2. Image A.M. Harvey.

Data were collected between December 2015 and March 2017 in the Kedumba Valley, an isolated section of approximately 130 km^2^ within the Warragamba Special Area of the Blue Mountains National Park, being part of the Greater Blue Mountains World Heritage Area in New South Wales, Australia. The horse population there was known to be small and geographically constrained since immigration and emigration are inhibited by natural physical boundaries, with Lake Burragorang to the south, high cliff faces to the north and east, and a river and steep terrain covered with dense bush to the west (Figure 1 and Figure 2). In the past, some population exchange has been known to occur when a low water level in Lake Burragorang allowed horses to cross to and from the other side, but this was not possible during the study period. Two dirt roads provided access to the valley at the northern and western borders, locked gates preventing their use by horses. No management interventions had occurred in the preceding eight years. This study was part of a larger investigation of the population ecology and welfare of free-roaming horses conducted over a 15 month period (National Parks and Wildlife scientific license 101626, WaterNSW access license D2015/128332).

#### 2.1.3. Initial Surveys

**Figure 3:**
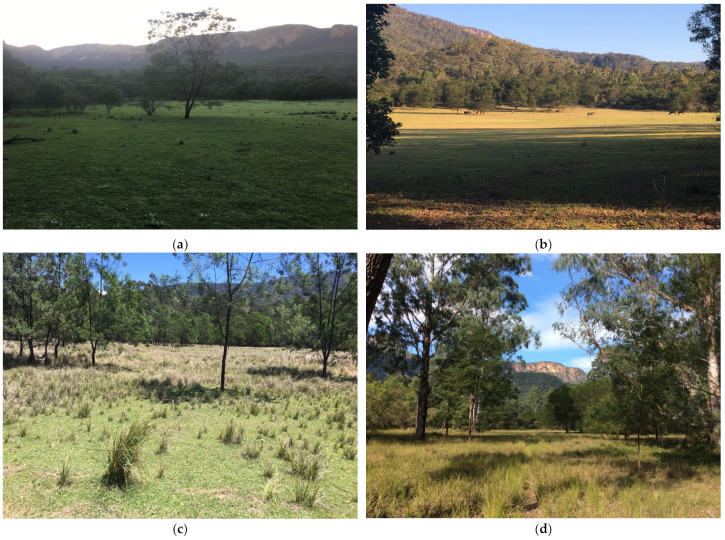

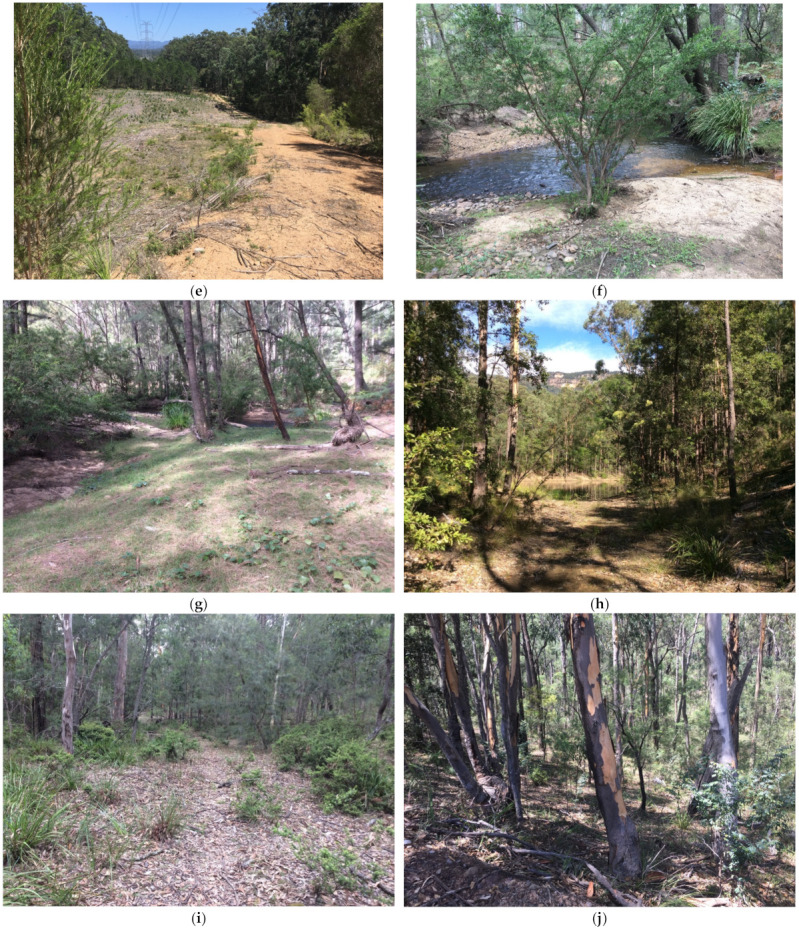

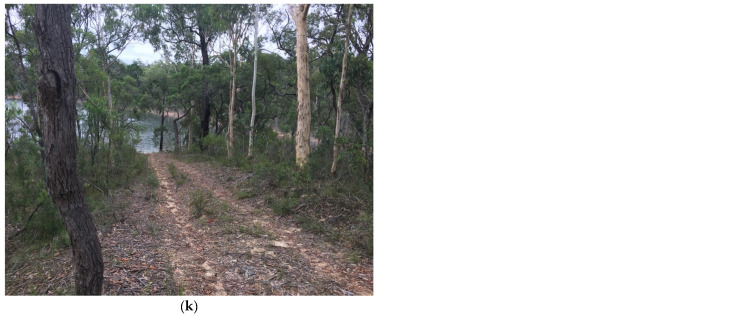
The eleven distinct areas of four main habitat types where horses resided. (**a**) Open grassland 1. (**b**) Open grassland 2. (**c**) Open grassland 3. (**d**) Open grassland 4. (**e**) Disturbed open woodland. (**f**) Riparian 1. (**g**) Riparian 2. (**h**) Woodland 1. (**i**) Woodland 2. (**j**) Woodland 3. (**k**) Woodland 4. Images A.M. Harvey.

**Figure 4:**
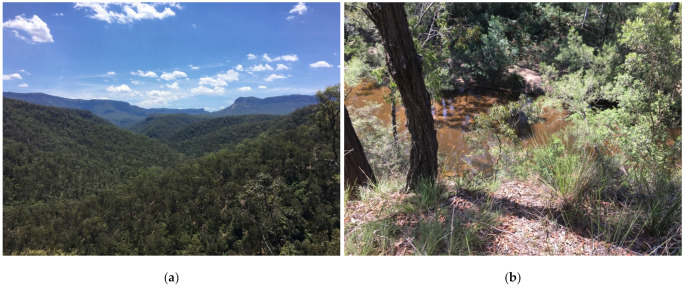
The predominant habitat and topography of Kedumba Valley: (**a**) Most of the region consisted of eucalyptus woodland across undulating and often steep terrain. (**b**) Most riparian corridors comprised steep cliffs making those sections of rivers inaccessible to horses. Images A.M. Harvey.

Extensive on-ground surveys were performed with a 4WD vehicle and on foot to become familiar with all regions of the valley in order to assess how resources varied spatially and to identify locations of horse tracks and/or faeces. Avenza maps (Avenza Systems Inc., Toronto, ON, Canada) were used on an iPad (Apple Inc., Cupertino, CA, USA) to navigate around the area and record GPS locations of the presence and abundance of horse faeces, horse sightings, water access points and habitat details. There was evidence of horses inhabiting eleven distinct areas dispersed within the valley, comprising four different habitat types; four small areas of open grassland with scattered woodland; two riparian areas; one area of disturbed open woodland situated below powerlines; and four woodland areas where water was accessible within 2 km. Figure 3 illustrates these eleven distinct areas. The remaining habitat comprised eucalyptus woodland and shrubs across undulating and often very steep terrain. Riparian corridors throughout these areas were surrounded by a high cliff, making them inaccessible to horses (Figure 4).

#### 2.1.4. Direct Observations

Direct observations were performed (by A.M.H.) on a total of 29 days, over 14 field trips of 1–3 days each, spread over the 15 month period. During each field trip, all eleven areas were surveyed, with direct observations occurring opportunistically whenever horses were sighted. The duration of direct observations depended mainly on how long horses stayed within sight, and so was not standardized. When horses stayed within sight for more than 1–2 min, they were observed using 10 × 42 binoculars (Bushnell Powerview FOV 293FT, Bushnell Corporation, Overland Park, KS, USA). A 900 m laser range finder was used to measure distances to horses, thereby enabling estimation of the maximum distances at which each welfare indicator could be assessed. Where possible, magnified photos and video footage (Canon EOS 70D Digital SLR with Canon EF100–400 mm lens) were taken of horses so that welfare indicators could be evaluated more closely at a later stage.

#### 2.1.5. Camera Trap Placement and Settings

Initial on-ground surveys and direct observations were used to identify suitable camera locations to maximise image capture of horses, for example, near drinking locations, pathways to a drinking location or prime grazing area, dirt tracks, horse trails, river crossing points, stallion faecal piles, and open grassland grazing areas where there was an abundance of horse faeces.

Forty-seven cameras (Bushnell Aggressor; Bushnell Corporation, Overland Park, KS, USA) were used throughout the study period, placed in a total of 58 different locations. Thirty-five cameras remained in the same location for the entire study period, five camera locations were changed slightly to improve the viewing angle of horses or to overcome false triggers, and seven cameras were moved to a different area to improve capture of horses in a particular area. Of the 58 different camera locations, 23 were in grassland habitats, 17 in woodland habitats, 13 in riparian habitats, and 5 in a disturbed open woodland habitat (Figure 1). Cameras were placed at a height of 70–110 cm, depending on the terrain. On dirt tracks and creek crossings, cameras were positioned with the aim of capturing the front of the horse approaching the camera, whilst on open grassland areas they were positioned to capture as much of the grassland area as possible. Initially all cameras were set to take still images only. During various periods over the whole study, 18 of the cameras (nine in open grassland, four in riparian habitats, four in woodland habitats and one in the disturbed open woodland habitat) were set to take hybrid video records, which comprised a still image followed by a video clip. Once triggered, videos ran for a specified duration; video clip duration was set at 5, 10, 15, 20, or 30 s. Camera settings were as follows: 14 M pixel image size or 1920 × 1080 video resolution; video sound on; capture number (i.e., number of still images taken in a sequence once the camera was triggered) ranged from one-to-three; camera time delay interval before responding to another trigger varied between 1 and 5 s; light-emitting diode (LED) control high, sensor level auto, night vision shutter speed medium, and camera mode 24 hrs. An identifying number for each camera, date, time and GPS co-ordinates, were entered into each camera. Camera locations were marked on Avenza maps to assist with relocating them. Each camera was checked, and batteries and secure digital (SD) cards changed, during each of the 14 field trips.

#### 2.1.6. Acquisition of Demographic Details and Identification of Individual Horses

Camera trap images were used together with resighted identification marks of horses under direct observation, in order to obtain unique identifying features for each horse, in addition to its sex, age category, and familial relationships. Horses observed early in the study period close together on multiple occasions, in the same geographical area, but distant (>1 km) from other horses were considered to constitute a distinct band.

A list of identifying features was made for each horse, including body coat colour, limb coat colour (if different from the body), mane and tail colour (if different from the body), facial markings (blaze, stripe, star, snip) and their size and shape, and limb markings (including the length the marking extended up the limb). An identification diagram was also completed for each horse to record the presence, size, and shapes of facial and limb markings. A unique identifier was then assigned to each horse.

#### 2.1.7. Assessment of Camera Trap Images and Videos

Images and video footage from all cameras over the 15 month study period were viewed and all ‘sightings’ where at least one horse could be identified were recorded. Each unique combination of horses captured within a duration of one second on a particular camera on a particular day constituted a separate sighting. From these data, a unique camera-day was defined as each camera-date combination where at least one horse was seen.

A stratified-random subset of camera-days from the full dataset of 2071 camera-days was used for detailed evaluation of whether individual horses could be identified and whether selected welfare indicators (Table 2) could be assessed. To do so, the study was divided into five 90-day time periods, broadly aligned with seasons (period 1: summer; period 2: autumn; period 3: winter, period 4: spring; period 5: summer) and for each camera method (still images or video), from each time period, one camera-day was selected from each camera that recorded sightings in the 90-day period, using computer-generated random numbers.

Within each selected camera-day, every observation event was assessed (by A.M.H.). An observation event was defined as a series of consecutive still images or video clips of the same horse on the same camera on the same day, with a maximum of a 3-min interval between consecutive images or video clips where the horse was visible. The observation event ended when the horse left the camera’s field of vision and did not return within 3 min. Thus, one horse could have multiple observation events on the same camera on the same day. Other horses could be in view for part or all of another horse’s observation event; each horse in view was treated as a separate observation event. Table 3 lists the details recorded for each observation event. For the purposes of this study it was only recorded whether or not it was feasible to assess each indicator, with regard to whether the relevant part(s) of the horse were captured at an appropriate distance to the camera, or for indicators such as gait evaluation and weakness, requiring dynamic records, whether enough of the horse was captured moving within video clips. Precise descriptions that enable the particular indicators to be categorized in terms of potential welfare compromise in each physical/functional domain will be provided in subsequent manuscripts.

Qualitative behaviour assessment (QBA) [42,46,54,55] was performed on video-observation-events, by an experienced observer (A.M.H.), using the fixed descriptors of ‘dull’, ‘relaxed’, ‘alert’, ‘apathetic’, ‘curious’, ‘anxious’, ‘playful’. If such assessment was not possible, the reasons were recorded. In fact, QBA scores have not been reported here because the present purpose was restricted to evaluating whether or not video-observation events captured enough of the ‘whole horse’ behaviour to enable a QBA to be conducted.

Observations of specific behaviours were also quantified. Behaviours were assigned to five main categories using a previously defined ethogram for free-roaming horses [13]. Categories were: locomotion (subcategorised as walking, trotting, cantering); standing resting; grazing; maintenance behaviours (including grooming, nursing, rolling, lying down, standing alert, drinking, urinating, defaecating); and social behaviours (included allogrooming, play behaviour, nuzzling, sexual interactions, and other affiliative and agonistic social interactions).

A behaviour event was defined as an occurrence or period in which an individual horse performed a particular behaviour without interruption. The duration of these behaviour events was recorded in terms of the number of images and/or times when the behaviour was observed. The end of a behaviour event occurred either when the horse left the camera’s field of view or changed to a different behaviour. Table 4 lists additional information recorded from each behavioural event.

These results were used to evaluate the relative proportions of the different behaviours captured by camera traps. For still images, the relative proportions of different behaviours were calculated separately, based on the number of images, and the period of time for which the same behaviour was observed (Table 4).

Social bonds were also analysed by reference to ‘close spatial proximity’ [60]. For the purposes of this study, this was defined for each observation event as occurring if other horses were present at the same time in any image or video frame in addition to the focal horse for that observation event, or in a subsequent image taken within one second of the previous image. Since close spatial proximity could occur concurrently with a range of other behaviours, proximity was analysed separately to other social behaviours. Furthermore, for less frequently performed social behaviours, such as allogrooming, play behaviour, nuzzling, sexual interactions, and other affiliative and agonistic social interactions, an ‘all occurrence’ method was used where the entire dataset of 2071 camera-days (still images and video clips pooled) was evaluated, and the behaviour recorded each time it was captured on images or videos.

All data were entered into standardized Excel 2011 spreadsheets (Microsoft, Washington, DC, USA) with separate spreadsheets for data from still images, from video, and for quantifying behavioural observations.

### 2.2. Data Analyses

Statistical analyses were performed using Stata (version 16.1; StataCorp, College Station, TX, USA). The numbers of images required for a horse to be identified via still-image observation events was assessed as time-to-event data using Kaplan–Meier survivor and failure functions [61], where the ‘failure’ event in this context was identification of the horse. This approach allowed inclusion of observation events where the horse was not identified. For these events, the record was right-censored at the maximum image number for the horse in that observation event. In time-to-event analyses, right-censoring is used when a subject leaves the study before an event occurs (in this case before the horse is identified). The Kaplan–Meier failure function value at a specified number of images is equivalent to the cumulative percentage of observation events where the horse was identified by that number of images if none-were right-censored. Survivor functions were compared using log rank tests, by day/night, habitat, horse coat colour, facial marking and limb marking categories. For horse-level factors, only observation events where the horse was identified were used. The same methods were used to determine the times at which the horse was identified for still-image observation events. Stata’s-*sts list*-and *sts test* commands were used.

Separately for each welfare indicator, proportions of observation events where an indicator could be assessed were compared between habitats and between methods (still images or video) using logistic regression, with both variables fitted simultaneously. Stata’s *logistic* command was used. For bilateral welfare indicators (e.g., wounds, limb pathology, and skin lesions), each observation event was classified as one of: could be assessed on both sides/one side/neither side. Then, separately for each welfare indicator, generalised ordered logit models were fitted using the *gologit2* command in Stata. Where the *p*-value for the proportional odds/parallel lines assumption was >0.05, proportional odds were assumed and ordered logit models were fitted using Stata’s *ologit* command. Likelihood ratio test *p*-values were used.

Reasons why welfare indicators could not be assessed were compared between habitats and between methods using the same approach as that for evaluating whether non-bilateral welfare indicators could be assessed (i.e., those welfare indicators that could be assessed without seeing both sides of the horse), with one exception. Blurred images were not an impediment in any of the video-observation events, so methods were compared using exact logistic regression with Stata’s *exlogistic* command. Habitat was not fitted, and sufficient statistics were used, rather than the other main statistical alternatives of conditional scores tests or conditional probabilities tests. For all analyses, observation events were treated as if they were statistically independent of each other after accounting for the variables fitted in each model.

## 3. Results

### 3.1. Demographic Details of the Population

Twenty-nine horses were identified in the population during the study period. These horses were distributed across five bands, with a total of 5 stallions, 16 mares, 3 fillies, 4 colts and one foal of unknown sex (Table 5).

### 3.2. Direct Observations

**Figure 5:**
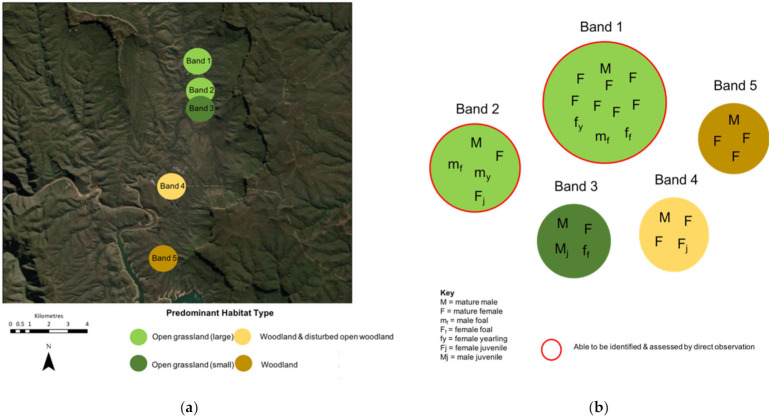
Illustration of (**a**) the geographical location and predominant habitat type of the different bands, and (**b**) band compositions. Only horses in Bands 1 and 2 that resided on larger open grassland habitats could be identified and assessed by direct observation. Horses in all other bands and habitats could only be identified and assessed with remote camera traps.

All individuals in Bands 1 (11 horses) and 2 (5 horses), who predominantly frequented open grassland habitats, were identified during the first two of the 14 field trips. The remaining 13 horses in the population were never identified by direct observations during the 15 month study. Figure 5 illustrates the band compositions, their habitats and whether or not they were directly observed.

Horses were observed and identified in open grassland habitats during all 14 field trips and in riparian habitats during 4/14 field trips. Horses were observed in woodland and disturbed open woodland habitats during 4/14 field trips, but only 3 horses were identified (on a single field trip) during observations in woodland habitats, and none were identified in disturbed open woodland. On all other occasions in the woodland and disturbed open woodland habitats, horses moved out of sight quickly, preventing their identification or assessment of welfare indicators.

**Figure 6:**
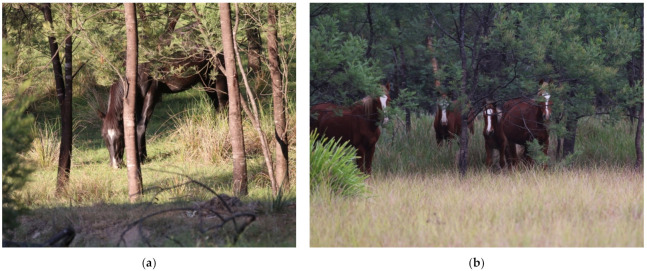

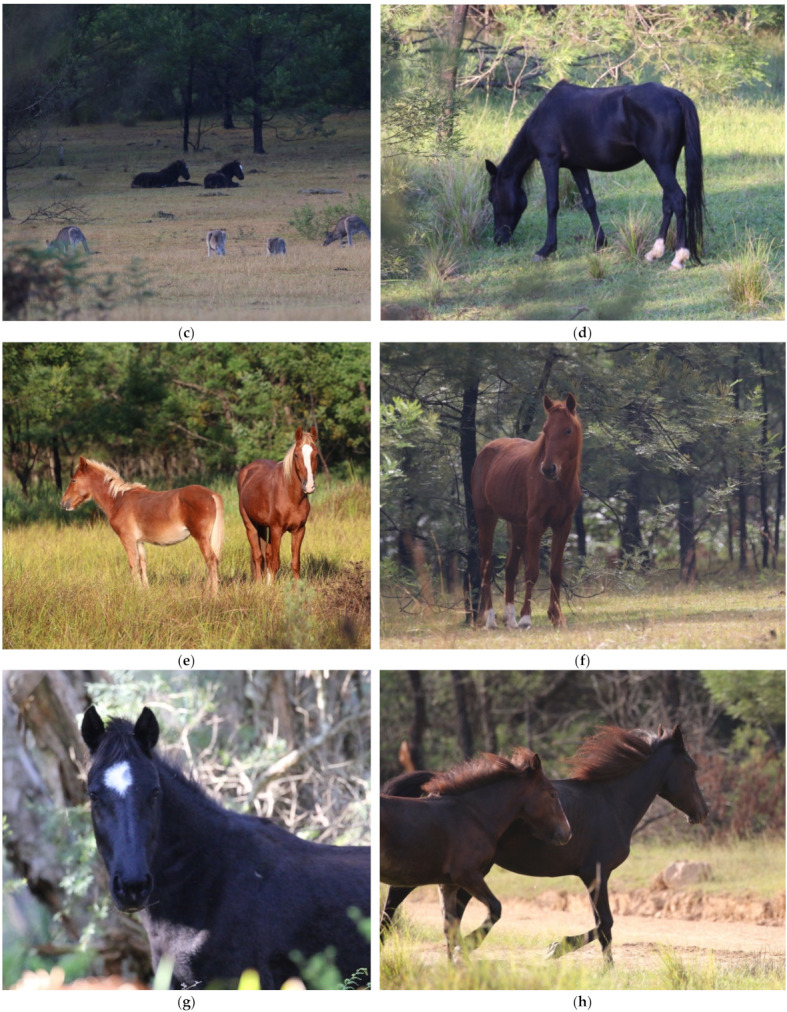

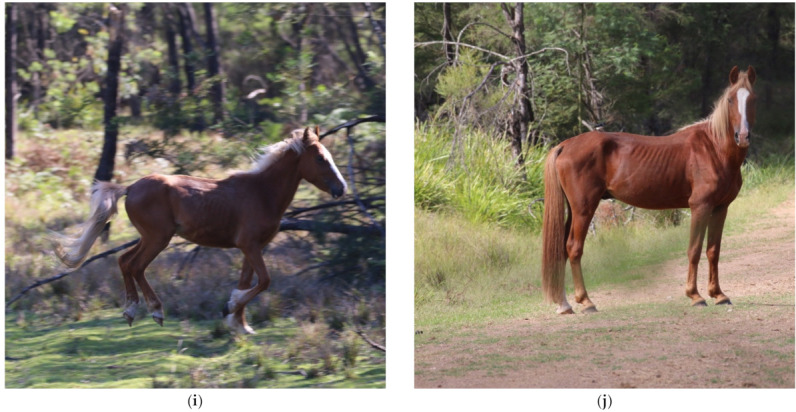
Examples of identification of horses and assessment of welfare indicators with direct observations (with 8–10× magnification), and some reasons for not being able to assess indicators. (**a**) Behaviour can be assessed but other indicators are obscured by vegetation. (**b**) Close spatial proximity with other horses can be assessed, but other indicators are obscured by vegetation. (**c**) At >100 m, behaviour is being observed without disturbing the horses, but the distance is too great to assess other welfare indicators. (**d**) At 100 m from the horse most welfare indicators can be assessed, but in this case the orientation of the horse prevents assessment of facial features and the right side of the horse. (**e**) At approximately 50 m, most welfare indicators can be assessed, but at this distance the observer will be seen by the horse, altering its behaviour. Distal limbs are obscured by vegetation. (**f**) Facial indicators can be assessed from approximately 50 m. (**g**) The detail of facial features is much greater <50 m to the horse but it is rare to be able to approach them so closely. (**h**) Trot is more readily assessed with direct observations. (**i**) Canter is more readily assessed with direct observations. (**j**) A good example of where most welfare indicators can be assessed on the right side of the horse, but at such a close distance behaviour is impacted by the observer’s presence. Photographs A.M. Harvey.

Welfare indicators could only be assessed directly in the 16 horses (Bands 1 and 2) in the larger open grassland habitats, the primary determinants of success being how long each horse remained in sight and how close it was to the observer. Proximity influenced whether or not individual horses could be identified, and which welfare indicators could be assessed (Figure 6). Using 10× magnification binoculars, the distances where specific attributes were observable were as follows: horses within approximately 150 m could be identified as individuals and their body posture and behaviour assessed; at approximately 100 m, all indicators other than facial indicators (i.e., facial grimace, blepharospasm, ocular discharge, nasal discharge, food pouching and quidding), and hoof condition could be assessed, although closer distances enabled more accurate assessment and detection of more subtle abnormalities; and within 50 m, facial indicators and hoof condition could be assessed. Photographs/videos were acquired with a 400 mm lens, which provided 8× magnification, enabling welfare assessments at similar distances to dynamic live direct observations with binoculars, but with the benefit of less hurried deliberation.

Although welfare indicators may be assessed at longer distances using more powerful magnification devices, the observer needs a clear line of sight, unobscured by trees and undulating terrain. In this study, the maximum such distance was approximately 300 m, which only occurred on one open grassland location occupied by Band 1. Horses were mostly observed from distances of 50–100 m, and so the facial indicators and hoof condition could not always be assessed. Horses in Bands 1 and 2, which were observed regularly, became more habituated to observer presence throughout the study. By the end of the study, all of their welfare indicators could be assessed for longer periods at distances of 20–30 m. In all other habitats, the unobscured line of sight was rarely greater than 20 m, but none of this sub-population of horses would allow such close proximity.

### 3.3. Camera Trapping

#### 3.3.1. General Statistics

Over the 15 month period, a total of 220,836 image/video files (a file constitutes a single image or video clip) were obtained. Of these, 42,925 (19%) image/video files contained horses whilst the remainder contained only other animals (e.g., macropods) or were false triggers due to wind-blown vegetation moving in front of the camera.

One or more horses were seen and identified via at least one camera on 428 days (95%) of the study period. For individual cameras, one or more horses were seen and identified on a mean of 9.5% of the camera’s days (range 0–45.9%). By habitat, of the cameras located in open grasslands, the corresponding average was 12.5% of the camera’s days (range 0–45.5%), which was similar to cameras located in disturbed open woodland (mean 14.4%; range 0–45.9%). In contrast, horses were seen and identified on lower proportions of days by cameras located in riparian (mean 6.8%; range 1.7–21.6%) and woodland habitats (mean 3.6%; range 0–21.2%).

In all, 199 camera-days were randomly selected from the 2071 camera-days that had detected horses, of which 158 days were still images and 41 were videos. Within the 158 camera-days of still images, there was a total of 538 observation events (range 1–23 observation events per camera day, mean 3.4, SD 3.44, median 2). Within the 41 camera-days of video clips, there was a total of 119 observation events (range 1–11 observation events per camera-day, mean 2.9, SD 3.85, median 1).

For still images, there was a range of 1–36 images per observation event (mean 4.2, SD 4.75, median 3) with the duration of observation events ranging from <1–257 s (mean 47, SD 113, median 4). For the 411 observation events with more than one image, the time between images ranged from <1–163 s (mean 14.5 secs, median 4.3, SD 25.76).

For videos, the duration of observation events ranged from 1 to 252 s (mean 29.3, SD 46.3, median 5). The most common total observation event durations were 5 s in 40/119 (33.6%), 10 s in 12/119 (10.1%), and 30 s in 38/119 (31.9%). Observation events comprised between 1 and 11 separate video clips with 26/119 (20.2%) involving more than one clip (mean 1.63).

Longer video clip durations resulted in the horse being visible for longer (Table 6), such that each additional 5 s added, on average, 3.1 s where the horse was visible. For the 5 s duration, the horse was usually visible for the entire 5 s, whereas, for the 10-, 15- and 30 s durations, the horse was visible for 50–61% of the time (Table 6).

#### 3.3.2. Identification of Individual Horses

Of the 29 horses in the study population, 27 (93%) had at least one randomly selected still image observation event, with the number of such events for each of those horses ranging from 2 to 33 (mean 14.9). Twenty horses (69%) had at least one randomly selected video observation event, with the number of observation events per horse ranging from 1 to 11 (mean 3.2).

For still images, the individual horse was identified in 405 of 538 (75%) observation events. In 62% (333/538; 95% CI, 58% to 66%) of observation events, the horse was identified from the first image. The Kaplan–Meier failure function (indicating successful identification) rose to 83% (95% CI, 78% to 87%) by five images. Increases with further images were only small. By 16 images, the Kaplan–Meier failure function was 93% (95% CI 86% to 97%). For the time until identification, the horse was identified immediately in 333/538 (62%) observation events. The Kaplan–Meier failure function was 70% by 10 s (95% CI, 66% to 75%) and 77% (95% CI, 73% to 82%) after 30 s.

**Figure 7:**
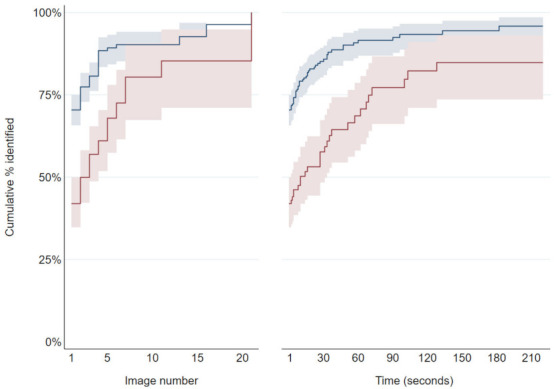
Cumulative percentages of observation events where the horse was identified by number of images (left-hand graph) and time from start of each observation event (right-hand graph) from still image observation events in the daytime (blue) and night-time (red). Kaplan–Meier failure functions are graphed; these are equivalent to cumulative percentages of observation events where each horse had been identified by the specified *x*-axis if no observation events had been right-censored. Shaded areas are point-wise 95% confidence intervals.

Identification of individual horses was more rapid with still images taken during daylight than with images taken at night (*p* < 0.001 for both number of images and time to identification). During the day, 70% of horses were identified with one image, and 88% of horses were identified after 4 images, whereas at night only 42% of horses were identified after one image, and 61% after 4 images (Figure 7). Patterns of time to identification were similar (Figure 7).

There were no substantial differences in the numbers of images or times to identification between horses with different coat colours or markings.

**Figure 8:**
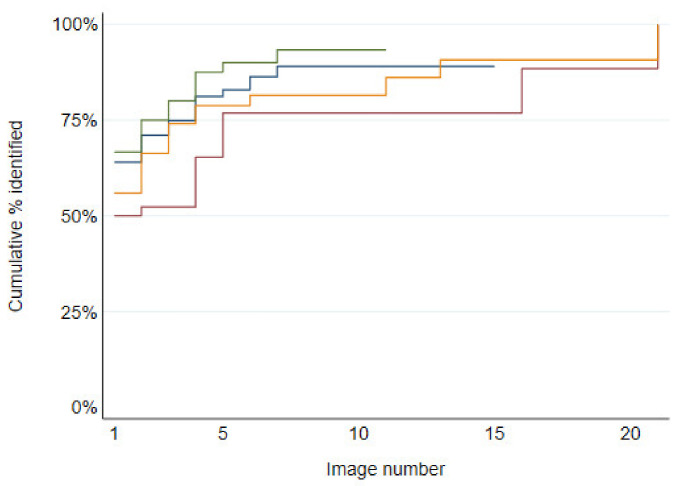
Cumulative percentages of observation events where the horse was identified by number of images from still image observation events in various habitats: riparian (green); open grassland (blue); woodland (orange); open disturbed woodland (red). Kaplan–Meier failure functions are graphed; these are equivalent to cumulative percentages of observation events where the horse was identified by the specified *x*-axis value if no observation events had been right-censored.

Of the 538 observation events, 300 occurred in open grassland habitats, 99 in riparian areas or at river crossings, 93 in woodland habitats, and 46 in disturbed open woodland habitats. Numbers of images until the horse was identified varied by habitat (overall *p* = 0.03), with the greatest numbers of images required for identification in observation events in open disturbed woodland habitats (Figure 8).

The most common reason for not being able to identify a horse in the first image was being unable to see facial or limb markings in the image (n = 31). Other reasons were only a small part of the horse being in the image, for example only the back, side, neck, shoulder or head side on (n = 24), the horse being too distant +/− at night (n = 5) and horse obscured by another horse or vegetation (n = 2).

**Figure 9:**
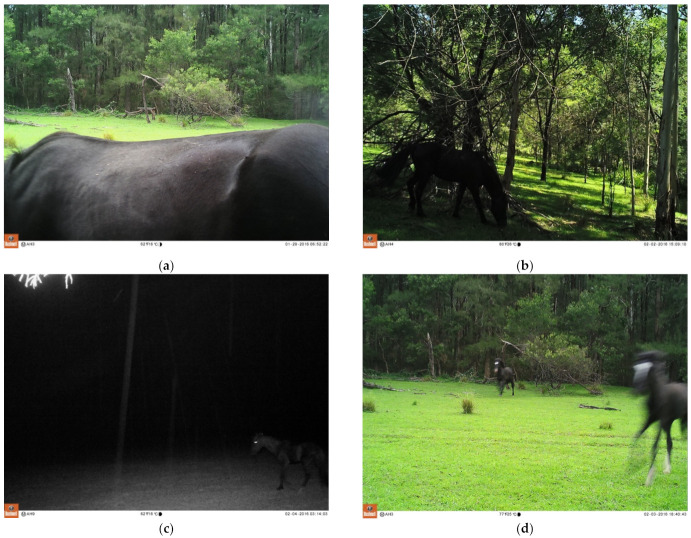

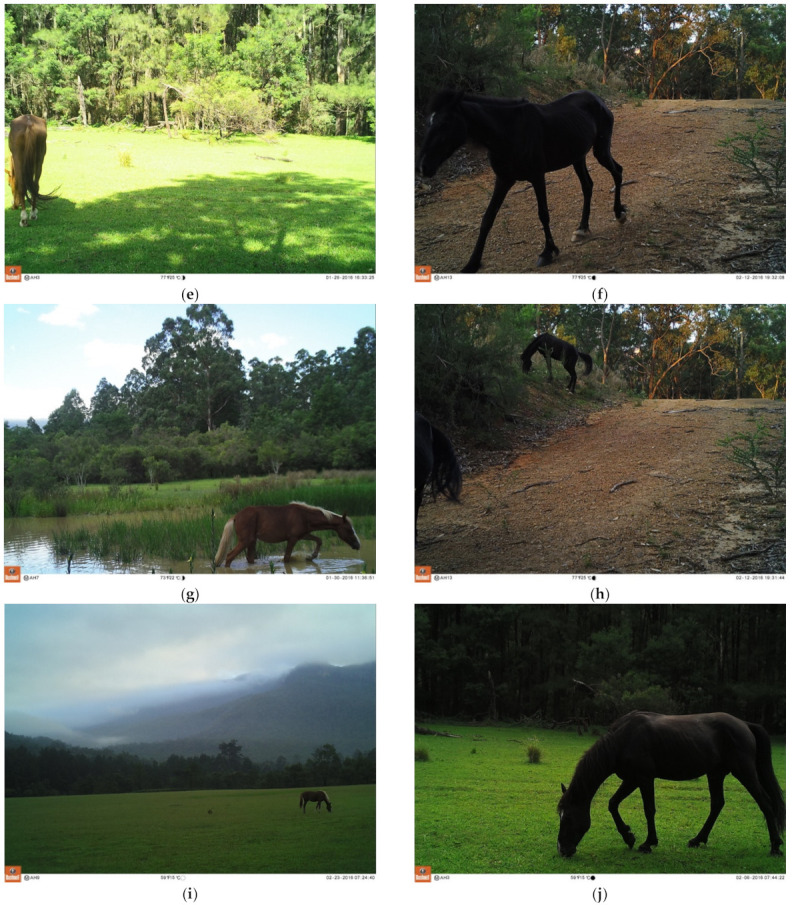

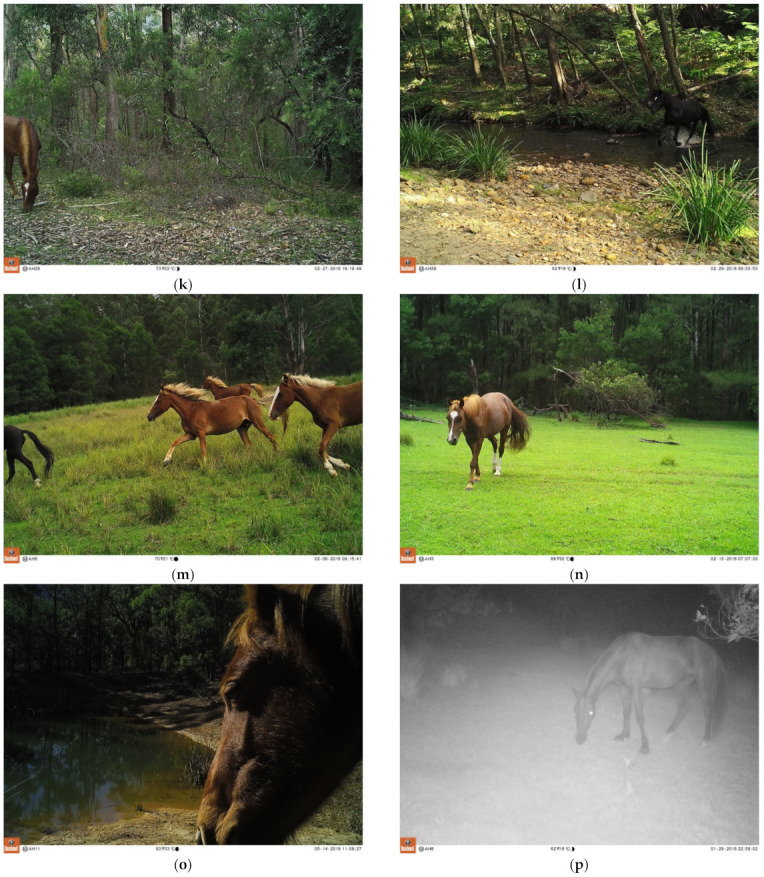

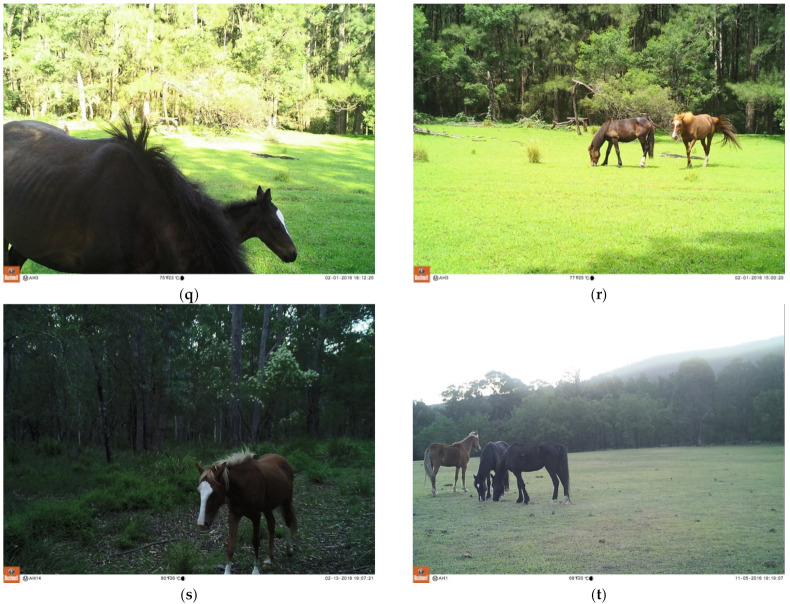
Examples of identification of horses, and assessment of welfare indicators with camera trap images, and common reasons for not being able to identify horses or assess indicators. (**a**) This horse was too close to the camera resulting in only the top half of the horse being in the image, therefore it was not possible to identify or assess welfare indicators other than body condition on this particular image. (**b**) Although this image was taken during daylight, shadowing from trees resulted in the image being too dark to identify the horse or welfare indicators. (**c**) A night-time image illustrating the difficulty in identifying horses or indicators in night-time images. (**d**) These horses’ distinctive markings make them identifiable, and it can be determined that both horses are cantering, however blurring of the image means no other welfare indicators are able to be assessed. (**e**) This horse can be identified from its colour and hindlimb markings, and it is evident that the horse is grazing, but no other welfare indicators can be assessed due to only the back of the horse being visible. (**f**) This horse can be identified, and it is evident that the horse is walking; body posture, body condition, hoof condition and presence of limb pathology can be assessed, but other welfare indicators are unable to be assessed as the image is too dark. (**g**) This horse cannot be identified on this single image as the shape of the blaze is not evident from the side-on image, and the limb markings are obscured by the water. Body condition and presence of wounds on the right side can be assessed. (**h**) The horse on the left cannot be identified as only the rump and tail are in the image, however it is enough to ascertain that the horse on the right is not alone. The horse on the right can be identified from its limb markings, and is observed to be grazing but the image is too dark and the horse too distant from the camera to be able to assess any other welfare indicators. (**i**) This horse can be identified from his colour, limb markings, sex, and length of tail suggesting he is juvenile. It is evident that he is grazing, but he is too distant from the camera to enable assessment of other welfare indicators. (**j**) A closeup image of a horse just at the perfect distance and orientation to the camera to enable both its identification and assessment of all welfare indicators on the left side. (**k**) This horse is identifiable from her colour and distinctive facial marking, and it is evident that she is grazing. An appreciation of body condition is possible although an accurate assessment of body condition score cannot be made with the limited proportion of the body captured in the image, and she is too distant to the camera to be able to assess facial indicators accurately. No other indicators are able to be assessed from this image. (**l**) This horse is identifiable from her facial marking, and it is evident that she is cantering. Body posture and body condition can be assessed, but she is too distant from the camera to enable assessment of other welfare indicators. (**m**) This image captures four horses from Band 2 cantering together and illustrates close spatial proximity between them all. Most welfare indicators can be assessed from the left side of the horse in the centre of the image, and a variable number of indicators can be assessed for the other horses due to distance from the camera or only half the horse being in the image. (**n**) This horse is at a good orientation to and distance from the camera to enable most indicators to be assessed on the left side. (**o**) A close up facial image enables assessment of facial indicators on the left side of this horse. (**p**) Although a night-time image, this horse is can be identified, and body condition and body posture assessed. (**q**) Only parts of the horses are visible in this image hindering assessment of welfare indicators, however the distinct facial marking on the foal enables his identification; facial indicators on the right side can be assessed in the foal, whilst body condition can be assessed in the mare. (**r**) This image shows close spatial proximity between two identifiable horses, with the horse on the left grazing and the horse on the right walking in a relaxed posture. Body condition, coat condition, body posture, limb pathology, presence of wounds on the left side, and, in the chestnut horse, some facial indicators on the left side can be assessed. (**s**) The orientation and distance of this horse from the camera enable most indicators to be assessed on the left side, except hoof condition since the front hooves are missing from the image. (**t**) This image shows close spatial proximity between three horses with the two on the right grazing and the chestnut on the left standing alert. Body posture and body condition can be assessed, but distance from the camera precludes assessment of other welfare indicators.

The reasons for not being able to identify a horse at all for a whole observation event were only a small part of the horse being in the image (n = 57), the images either being too dark, blurred and/or the horse too distant (n = 31), being unable to see facial or limb markings in the image (n = 25), and the images captured at night (n = 24). Some examples are shown in Figure 9.

For camera trap video, horses were identified in 72% (86/119) of observation events, and when this was the case, it was always possible to identify horses at the beginning of the observation event. Reasons for the horses not being identifiable were only a small part of the horse being in the video (n = 18), the horse being too far from the camera (n = 12), and an inability to see facial markings (n = 1).

### 3.4. Assessment of Welfare Indicators

The welfare indicators that can be assessed most frequently on both still camera trap images and video, were body condition score (73% and 79% of observation events, respectively), body posture (76% for both), coat condition (42% and 52%, respectively), and whether or not the horse was sweating excessively (42% and 45%, respectively) (Table 7). Coat condition was the only welfare indicator where there was evidence of differences between still images and videos, where it could be assessed more often via video (Table 7).

Body condition score was less likely to be assessable in open grassland (67% of observation events) than in other habitats (80% to 85%; overall *p*-value for habitat adjusted for method <0.001). Percentages of observation events where body posture could be assessed were similar for each habitat (74% to 78%) and the *p*-value for differences between habitats was high (0.860). Hoof condition, coat condition, sweating, and facial grimace were all less able to be assessed in open grassland habitats (for each, *p* < 0.001).

The most common reasons for not being able to assess particular welfare indicators were images/videos being captured at night and the horse being too distant from the camera, such as in open grasslands (Table 8). Assessment of hoof condition was prevented when hooves were obscured by vegetation, mud, or water. Although night-time commonly prevented a range of welfare indicators from being identified, body condition score (68% and 75%, respectively) and body posture (75% for both) could be assessed as frequently in night-time as in daylight observation events, whereas coat condition could only be assessed in 22% of night-time observation events compared with 51% of those in daylight. Some examples are shown in Figure 9.

An additional seven welfare indicators were assessed only in video observation events as we considered a priori that it would never be possible to assess these with still image observation events. Of these, the indicators that were able to be assessed most frequently were presence or absence of weakness (66%), qualitative behavioural assessment (60%), presence or absence of shivering (51%) and gait at walk (50%, Table 7). Gait at trot and canter could not be assessed in any observation events, but respiratory rate and effort could be assessed in 36% of video observation events (Table 7). For assessments of hoof condition and facial grimace in video observation events, numbers were sufficient to assess the usefulness of additional video clips within the observation event. For 15 and 20 observation events, respectively, these indicators could not be assessed in the first video clip nor in any of the further 1 to 10 video clips.

Gait at walk (overall *p* = 0.010), respiratory rate and effort (overall *p* = 0.006), and shivering (overall *p* = 0.018) were less likely to be assessable in open grassland habitats than in woodlands. The most common reasons for not being able to assess video-specific welfare indicators were: only a small part of the horse being in the camera’s field of view (n = 28); the horse not moving substantially during the video recording (n = 18); the horse being too distant from the camera (n = 16); and the horse not being in the camera’s field of view for long enough (n = 11).

### 3.5. Quantifying Behavioural Observations

From the random selection of camera days, 601 behaviour events were identified on still images, the specific character of which could be identified in 560/601 (93%) of these (Table 9). On video clips, 213 behaviour events were identified, the specific character of which could be identified in 178/213 (84%) of these. Behaviours were unidentifiable if the horse was still and the head and/or limbs were not in the camera’s field of view.

The most commonly observed behaviours were grazing and walking, with grazing occupying a larger proportion of time, being 60% when based on duration of time taken from still images, 43% when derived from number of still images, and 51% when timed from video clips (Table 9). Social behaviours were rarely observed, with only two such events detected, namely, a horse sniffing a stallion faecal pile, and one horse sniffing another.

In addition, close proximity of the focal horse to other horses was recorded in 236/657 (36%) of observation events [202/538 (38%) for still image and 34/119 (29%) for video observation events]. Using the ‘all occurrence’ approach to further evaluate uncommonly observed behaviours, specific social interactions between two or more horses were only recorded on 39 occasions within the full dataset of 42,925 image/video files. These interactions were mostly affiliative (35 affiliative interactions including allogrooming, being herded or herding other horses, trotting, cantering or galloping with other horses, frolicking, nuzzling, playing, physical contact with another horse whilst walking), with three occasions of reproductive behaviours (mating/being mated, flehmen response, winking), and one agonistic event (chasing another horse).

## 4. Discussion

This paper describes, for the first time, camera trapping methodology that can be successfully applied across a range of habitats in order to identify individual horses, and to non-invasively measure or observe a range of animal-based welfare indicators. Furthermore, it demonstrates the advantages and limitations of this methodology.

Methodological information is often lacking in camera trap publications [62]. We have sought to describe methods in sufficient detail to enable other researchers to easily replicate them. Further, as a result of this study we can make recommendations for others wishing to use camera trapping for this purpose. Firstly, extensive ground surveys to facilitate precise and strategic camera placement are key to optimising image quality and detection of horses at appropriate angles and distances from the camera in order to both identify individual horses and assess a range of welfare indicators. Deploying cameras on tracks, grazing areas, and drinking locations within the same region assists in capturing the full range of listed welfare indicators, including a wider range of behaviours. Secondly, the choice of camera settings is important to enhance the data obtained whilst also minimising battery usage and SD card storage. We recommend using an image capture number of one (i.e., the number of still images taken in a sequence once the camera is triggered), and a camera time delay interval of 1 s for cameras placed on tracks where horses may pass quickly, and 3 s for cameras overlooking grazing or drinking locations. For videos, a duration of 10 s is recommended. Other settings recommended are as described in the Materials and Methods. Whether or not to use both day-time and night-time settings is dependent on the precise purpose of the study; although fewer horses and welfare indicators can be identified on night-time images, meaningful information can still be obtained. Use of video will increase the range of welfare indicators that can be assessed, but does significantly increase battery usage, SD card storage, and the time required for processing and analysing the data. Whilst there are strategies for increasing battery power (e.g., solar panels) and storage (higher memory SD cards), these can be costly, and despite security measures, vandalism and theft can be an issue. We therefore recommend prioritising use of video in habitats where horses cannot be reliably observed directly, in regions where repeated capturing of non-target species is less likely, and where cameras are able to be checked within a 1 to 2 month period. In other situations, still images may suffice and complement direct observations.

In woodland habitats, direct viewing of horses is challenging and relying on this would have significantly underestimated the population size in this study. A major advantage of camera trapping is the ability to identify individual horses in any habitat. Furthermore, demographic information such as herd size and reproductive rates varied spatially and across different habitats, so such data obtained from direct observation alone would not have represented the whole population. Understanding population dynamics and the processes that influence them is critical for management of populations. In Australia, there are few demographic studies on wild horses [5,10,16], particularly within woodland habitats [17,18]. In south-east Australia, although free-roaming horses commonly reside in woodland habitats (eucalyptus forests) and undulating and often steep terrain, population and demographic data for horses in these habitats are lacking. Our study suggests that utilising camera trapping methodology could address some of these knowledge gaps.

Practical limitations in the frequency with which direct observations can be performed is another challenge in assessing the welfare of any free-roaming wild species. Both the severity and the duration of welfare impacts are important in assessing welfare [1,20,21,22,23,24], so repeated assessments are advantageous. Beneficially, the continuous collection of data by camera traps permits more frequent assessments to be made. Further advantages are that the horses are not disturbed, and are captured close-up, thereby enabling a range of welfare indicators to be assessed and informative behaviours to be quantified, even in horses that cannot be observed directly.

To date, published studies have predominantly used invasive measures of health that require physically capturing the animal to perform procedures such as physical examination and blood collection (reviewed by [63]). Whilst these measures are informative, if available, it is preferable to use the least invasive methods for welfare assessments. Our study shows that a wide range of indicators of different aspects of welfare can be evaluated non-invasively using camera traps, and for some horses also by distant direct observations.

Twelve indicators of welfare aligned with the first four domains of the Five Domains Model, were able to be assessed with equal frequency on both still images and video, with an additional five indicators able to be assessed on video. The most practically measurable and reliable animal-based welfare indicators able to be detected were: in Domain 1 (Nutrition), body condition score; in Domain 2 (Physical Environment), presence of sweating or shivering; in Domain 3 (Health), body posture, coat condition, gait at walk, and presence/absence of weakness; and in Domain 4 (Behavioural Interactions), qualitative behavioural assessment and assessment of close spatial proximity for evaluation of social bonds. Spatial proximity of horses may have been overestimated on grasslands due to the greater field of vision of cameras in this habitat, compared to woodlands. In future studies, we therefore recommend defining close spatial proximity as animals standing within two body lengths of each other [60], rather than animals simply being in the same image or video frame. Facial indicators and hoof condition could only be assessed infrequently due to the need for clear close-up images/videos of these body parts, the correct angle of view and an absence of obscuring vegetation. Some indicators were less likely to be detectable in open grassland habitats than in other habitats, because the horses were often further away from the camera in grassland habitats. Additionally, for bilateral indicators, most commonly only one side of the horse could be assessed.

Quantification of specific behaviours was also achievable by evaluating the proportions of time that each behaviour was captured, or the proportion of still images demonstrating a particular behaviour. Since camera traps only capture the behaviour being performed at the precise time that the horse is within camera view, these proportions do not necessarily accurately reflect continuously recorded time budget behaviours [3,5,13,14]. Camera location may also bias the behaviour detected, e.g., grazing is more likely to be detected on open grassland and walking more often captured on tracks. However, if cameras remain in the same location over time, useful information may be obtained regarding temporal changes in the proportions of different behaviours being performed.

Despite the many advantages of remote camera traps, there are limitations that need to be considered when interpreting data. Information can only be collected when horses are in the camera’s field of view, which is heavily dependent on camera placement and the number of cameras deployed. Problematically on still images, it can be difficult to assess the motivation for a behaviour, and whether it reflects a positive or negative mental state (Domain 5). For example, rolling can be both a maintenance behaviour [13,44] and an indication of abdominal pain [45,59]; lying in lateral recumbency is commonly linked to rest [13,44], but can also be due to abdominal pain [45,59]; and rubbing/scratching is a normal maintenance behaviour [13,44], but when performed excessively can be an indicator of pruritus [64,65]. Similarly, although some features of ‘facial grimace’ [48,52] could sometimes be identified on still images, the significance of this may be over interpreted when based on a single still image. When these events are observed directly or with video, additional indicators and the context of the behaviour usually assist interpretation.

## 5. Conclusions

As far as the authors are aware, this is the first study that describes in detail a remote camera trapping methodology that enables identification of individual free-roaming wild horses across a range of habitats and the assessment of an extensive range of animal-based welfare indicators. Camera trap images and video provided valuable information about the horses, particularly those that could not be sighted regularly, sighted for a long enough duration, or approached closely enough to enable direct assessment of welfare indicators, as was the case in woodland habitats.

The next phases of this research include applying the same methodology to larger populations across different geographical areas, in addition to incorporating these methods into a welfare assessment protocol to objectively evaluate how the welfare of wild free-roaming horses varies spatially and temporally. The described methodology can also form the basis of applications to other species.

## Figures and Tables

**Table 1:** The Ten-Stage Protocol for assessing the welfare of non-captive wild animals [1].

Acquire an understanding of the principles of Conservation Welfare. Acquire an understanding of how the Five Domains Model is used to assess welfare status. Acquire species-specific knowledge relevant to each Domain of the Model. Develop a comprehensive list of potential measurable/observable indicators in each physical domain, distinguishing between welfare status and welfare alerting indices. Select a method or methods to reliably identify individual animals. Select methods for measuring/observing the potential welfare indices and evaluate which indices can be practically measured/observed in the specific context of the study. Apply the process of scientific validation for those indices that can be measured/observed and insert validated welfare status indices into the Five Domains Model. Using the adjusted version of the Model that includes only the validated and practically measurable/observable welfare status indices, apply the Five Domains grading system for grading welfare compromise and enhancement within each Domain. Assign a confidence score to reflect the degree of certainty about the data on which welfare status has been graded. Including only the practically measurable/observable welfare alerting indices, apply the suggested system for grading future welfare risk within each domain.

**Table 2:** List of selected welfare indicators in each domain.

Domain	Animal-Based Indices	
	Still Images and Video	Video Only
1. Nutrition	Body condition score	
2. Physical Environment	Sweating	Shivering
	Wet from rain	
	Huddling together with other horses	
3. Health	Body posture	Gait at walk
	Hoof condition	Gait at trot
	Coat condition	Gait at canter
	Wounds or other injuries	Weakness
	Limb pathology	Respiratory rate and effort
	Skin lesions	
	Nasal discharge	
	Ocular discharge	
	Blepharospasm	
	Quidding	
	Food pouching	
	Facial grimace	
4. Behavioural Interactions	Specific quantifiable behaviours (feeding, resting, maintenance, locomotory and social behaviours)	Qualitative assessment of behaviour (dull, relaxed, alert, apathetic, curious, anxious, playful)

**Table 3:** Details recorded from each observation event.

Date, method (still image vs. video), camera number Identification of the individual horse when possible, if not recorded as unidentified Number of images of video clips in the observation event (and video duration setting) Start and finish time of the observation event Number of images (or for video, time) until the horse identity was determined Reason for being unable to identify an individual at all Whether the image or video was taken in the daylight or at night For each welfare indicator listed in Table 2, whether it was able to be assessed or not, and for those indicators whose status can differ between left- and right-hand sides of the same horse, whether they could be assessed on the left, right, or both sides Reason(s) why any of the welfare indicators could not be assessed Where there were two or more video clips within an observation event, each video clip was also individually assessed for all of the above

**Table 4:** Information recorded from each behavioural event.

The identifiable behaviour (or recorded as unidentifiable and reasons for that) Number of images per behaviour (for still images) Start and finish times of the behaviour (for still images this was recorded as the time of the first and last images in a series of consecutive images in which the behaviour was captured) The habitat in direct field of view of the camera (i.e., grassland, dirt track, riparian, river crossing, waterhole, horse track through woodland, rest area beneath trees, woodland clearing)

**Table 5:** Details of the population and unique identifying features.

Unique Identifier ^1^	Sex ^2^	Age Group	Reproductive Status	Coat Colour	White Facial Markings	White Limb Markings ^3^
1A	M	Mature	Band stallion	Black	Star and snip	None
1B	F	Mature	Foaled year 2	Chestnut	Star and stripe	BLH socks
1C	F	Mature	No foal	Chestnut	None	BLH and RF fetlocks
1D	F	Mature	No foal	Black	Small star	BLH pastern spots
1E	F	Mature	No foal	Black	None	BLH and LF pastern
1F	F	Mature	Yearling (1G) at foot	Black	Large star	BLH fetlocks
1G	F	Yearling	Yearling of 1F	Brown	Blaze	BLH socks
1H	F	Mature	Foal (1K) at foot	Black	None	BLH pasterns
1I	F	Mature	Foal (1J) at foot	Dark bay	Star and stripe	BLH fetlocks and LF pastern
1J	M	Foal	Foal of 1I	Black	Large stripe	LH pastern, small spot medial RH pastern
1K	F	Foal	Foal of 1H	Black	Small star and snip	None
1L	Unknown	Foal	Foal of 1B (year 2)	Bay	Star and snip	None
2A	M	Mature	Band stallion	Flaxen chestnut	Blaze	LH stocking
2B	F	Mature	Foal (2C) at foot	Flaxen chestnut	Blaze	None
2C	M	Foal	Foal of 2B	Flaxen chestnut	Blaze	None
2D	M	Yearling	Dam unknown	Flaxen chestnut	Thick blaze	BLF socks, LH stocking
2E	F	Juvenile	No foal	Black	Large star and stripe	LF pastern, BLH fetlocks
3A	M	Mature	Band stallion	Brown	Stripe	BLH socks
3B	F	Mature	Foal (3C) at foot	Black	Star and stripe	BLH and RF pasterns
3C	F	Foal	Foal of 3B	Black	Large stripe	BLH and LF fetlocks
3D	M	Juvenile	Colt with another stallion in Band	Flaxen chestnut	Blaze	LH sock
4A	M	Mature	Band stallion	Black	None	LH fetlock, RF coronet
4B	F	Juvenile	No foal	Black	Very small star	RH fetlock, LF pastern
4C	F	Mature	No foal	Black	Interrupted stripe	LH pastern, RH coronet
4D	F	Mature	No foal	Chestnut	Stripe	RH fetlock
5A	M	Mature	Band stallion	Black	Star	LF pastern, BLH fetlocks
5B	F	Mature	No foal	Chestnut	Very small star	None
5C	F	Mature	No foal	Black	Large star	LH fetlock, RH coronet
5D	F	Mature	No foal	Black	Large star	LH fetlock, RF coronet

^1^ Number = band number; Letter = sequential letter within band. ^2^ M = male; F = female. ^3^ LH = left hindlimb; RH = right hindlimb; LF = left forelimb; RF = right forelimb; BLH = bilateral hindlimb; BLF = bilateral forelimb.

**Table 6:** Video clip durations and the period during which the horse was visible in the video clip.

Video Clip Duration Setting (Seconds)	Number of Video Clips	Number of Video Clips That a Horse Was Visible for Full Video Duration	Mean Period during Which a Particular Horse Was Visible in Video Clip (Seconds)
5	110	106 (96%)	4.9
10	20	10 (50%)	7.3
15	18	11 (61%)	11.2
20	6	1 (17%)	8.3
30	40	22 (55%)	20.6

**Table 7:** Welfare indicators assessed with camera trap still images and video.

Welfare Indicator	Able to Be Assessed with:		*p*-Value for Comparison of Ability to Assess Indicator between Still Image and Video Observation Events ^1^
	Still Images: Number of Observation Events (% of 538 Observation Events)	Video: Number of Observation Events (% of 119 Observation Events)	
Body posture	411 (76%)	91 (76%)	0.972
Body condition score	392 (73%)	94 (79%)	0.237
Coat condition	225 (42%)	62 (52%)	0.053
Sweating	226 (42%)	54 (45%)	0.587
Facial grimace	102 (19%)	20 (17%)	0.441
Hoof condition	92 (17%)	25 (21%)	0.382
Wounds:			0.227
Both sides could be assessed	24 (4%)	8 (7%)	
Only one side could be assessed	198 (37%)	49 (41%)	
Limb pathology:			0.611
Both sides could be assessed	144 (27%)	40 (34%)	
Only one side could be assessed	36 (7%)	0 (0%)	
Skin lesions:			0.538
Both sides could be assessed	23 (4%)	7 (6%)	
Only one side could be assessed	180 (33%)	42 (35%)	
Nasal discharge:			0.604
Both sides could be assessed	21 (4%)	15 (13%)	
Only one side could be assessed	89 (17%)	13 (11%)	
Ocular discharge and blepharospasm:			0.550
Both sides could be assessed	17 (3%)	15 (13%)	
Only one side could be assessed	107 (20%)	14 (12%)	
Quidding or food pouching:			0.220
Both sides could be assessed	4 (0.7%)	5 (4%)	
Only one side could be assessed	80 (15%)	8 (7%)	
Gait at walk	NA ^2^	60 (50%)	NA
Gait at trot	NA	0 (0%)	NA
Gait at canter	NA	0 (0%)	NA
Weakness	NA	78 (66%)	NA
Respiratory rate and effort	NA	43 (36%)	NA
Shivering	NA	61 (51%)	NA
Qualitative behavioural assessment	NA	71 (60%)	NA

^1^*p*-value for method after adjustment for habitat. ^2^ NA: not assessed-we considered a priori that it would never be possible to assess these indicators in still image observation events.

**Table 8:** Reasons for being unable to assess welfare indicators on camera trap still images and video.

Reason for Being Unable to Assess Welfare Indicators	Still Images: Number of Observation Events (% of 538 Observation Events)	Video: Number of Observation Events (% of 119 Observation Events)	*p*-Value for Comparison of Reason for Being Unable to Assess Welfare Indicator between Still Image and Video Observation Events ^1^
Night-time images	172 (32%)	31 (26%)	0.345
Only top half of horse in the camera’s field of view	96 (18%)	26 (22%)	0.273
Only front of horse in the camera’s field of view	26 (5%)	1 (1%)	0.022
Only back of horse in the camera’s field of view	59 (11%)	20 (17%)	0.090
Only head in the camera’s field of view	35 (7%)	2 (1%)	0.020
Obscured by another horse	13 (2%)	1 (0.8%)	0.213
Hooves obscured by vegetation, mud or water	129 (24%)	18 (15%)	0.037
Horse too distant from the camera	178 (33%)	36 (30%)	0.845
Image too dark	190 (35%)	7 (6%)	<0.001
Image blurred	57 (11%)	0 (0%)	<0.001 ^2^

^1^ Likelihood ratio test *p*-value for method after adjustment for habitat. ^2^ Exact *p*-value for method; no adjustment for habitat.

**Table 9:** The number and durations of identifiable behaviours calculated from camera trap still images and videos.

Behaviour	Number of Behaviour Events from Still Images (n = 560)	Number of Behaviour Events from Video (n = 178)	Number (%) of Still Images (n = 2159 Images ^1^) with This Behaviour	Duration (%) of Time from Still Images (n = 36,711 s ^1^) with This Behaviour	Duration (%) of Time from Video (n = 1683 s ^1^) with This Behaviour
Locomotion total	341	83	902 (42)	6916 (19)	465 (28)
Walking	325	83	872 (40)	6900 (19)	465 (28)
Trotting	8	0	14 (<1)	8 (<1)	0
Cantering	8	0	16 (<1)	8 (<1)	0
Standing resting	56	31	276 (13)	7669 (20)	299 (18)
Grazing	148	56	938 (43)	22,025 (60)	851 (50)
Maintenance behaviours	13	7	39 (2)	99 (<1)	65 (4)
Social behaviours	2	1	4 (<1)	2 (<1)	3 (<1)

^1^ Total numbers are for pooled behaviour events where the behaviour was identified.

## Data Availability

The data presented in this study are available on request from the corresponding author. The data are not publicly available due to additional ongoing analysis.
